# Airway Smooth Muscle and Asthma

**DOI:** 10.3390/cells12060882

**Published:** 2023-03-12

**Authors:** Steven An, Dale D. Tang

**Affiliations:** 1 Rutgers Institute for Translational Medicine and Science, New Brunswick, NJ 08091, USA; 2 Department of Molecular and Cellular Physiology, Albany Medical College, Albany, NY 12208, USA

Airway smooth muscle (ASM) was first described in 1804 by Franz Daniel Reisseisen (as related by Otis (1983)) [1], and the “*irritability*” of ASM and its potential contribution to asthma was considered by Henry Hyde Salter in 1868 [2]. For many decades, this muscle layer that wraps circumferentially around the pulmonary airways did not receive much attention in respiratory mechanics and, at one point, was posited as a vestigial organ of “*no useful function in the lung*” [3,4], unlike the homeostatic regulation of blood flow or peristalsis dictated by the smooth muscle surrounding the lumen of blood vessels or gastrointestinal track.

Subsequently, it became clear that ASM is a pivotal cell regulating bronchomotor tone, airway caliber, and ventilation distribution in the lungs [5]. There is also widespread agreement that the principal mechanism of the increased airway resistance to airflow in asthma is, in part, due to actively constricted ASM, as well as increased ASM mass.However, despite major advances in asthma research, the underlying basis for enhanced ASM shortening and/or increased muscle mass is not well-defined. The mechanistic link between mechanical endotypes of airflow obstruction (due to ASM shortening/mass) and the immune inflammatory responses that characterize the clinical phenotype of asthma remains equally unclear.

This Special Issue of *Cells* is devoted to many aspects of the structural and functional changes (mechanical endotypes) associated with airflow obstruction in asthma. It begins with two reviews that collectively provide a balanced, state-of-the-art view of various aspects of this heterogenous lung disease. Habib et al. focus on the contribution of inflammation-dependent and inflammation-independent mechanisms in asthma pathogenesis, and also consider clinical phenotypes, including previously identified and newly emerging traceable substances (biomarkers) that may drive the progression toward distinct asthma endotypes. Borkar et al. provide a network perspective of the genomic and non-genomic effects of sex steroids in asthma pathobiology, focusing on the sex differences as a non-modifiable risk factor that may bias the function of immune and resident structural cells in the lungs at the gene, epigenetic and molecular levels. In sum, both reviews offer new opportunities in the current era of precision medicinefor better disease phenotyping and endotyping, which could translate into advances in asthma diagnosis, classification, and individualized treatments.

Patients with asthma typically experience periodic or persistent decreases in airflow from bronchospasm, and virtually all patients exhibit an exaggerated bronchoconstrictive response to exogenously administered agents such as histamine and methacholine. Despite advances in treatment, asthma remains poorly controlled. In the treatment of obstructive lung diseases, the use of combination long-acting muscarinic antagonist (LAMA) and long-acting β_2_-agonist (LABA) elicits superior bronchodilation in patients with COPD than that achieved with the administration of LABA or LAMA alone. As such, there is a new push to develop a small, single molecule with dual action (MABA). Navafenterol (AZD8871) is one such pharmacological agent, developed by AstraZeneca. In a short communication, Jude et al. applied a pre-clinical model of human precision cut lung slices (hPCLS) to assess the β_2_-adrenceptor agonism of navafenterol and its bronchoprotective effects against non-muscarinic spasmogens, i.e., the histamine and thromboxane implicated in asthma. The approach used in this study should lead to the better characterization of novel drugsthat are in the pipeline as a potential treatment for asthma, as well as their mechanistic actions.

In asthma, the key end-effector of acute airway narrowing is the contraction of the ASM cell driven by myosin motors exerting their mechanical effects within an integrated cytoskeletal scaffolding [5]. In this Special Issue, Wang et al. revisit an original purification method for myosin and detail a new approach that yields purified myosin, complexed with or without its associated regulatory proteins, including myosin light-chain kinase and myosin light-chain phosphatase. This is significant because it has the potential to modulate and visualize the structure–activity relationship of the contractile apparatus in ASM as they relate to the mechanical endotypes of asthma.

ASM contracts and shortens in response to a wide range of endogenous and exogenous agonists. ASM also proliferates and migrates upon alterations in external and internal environments, which are critical to lung development, tissue homeostasis, and airway remodeling, a key feature of asthma. The fundamental mechanisms that regulate ASM migration and proliferation are not entirely elucidated. Three original research articles in this Special Issue tackle these questions head-on, considering both classical and non-classical regulatory pathways.

Smooth muscle myosin II is traditionally thought to localize solely in the cytoplasm and regulates cell migration by affecting stress fiber formation, focal adhesion assembly, and retraction of the rear. Wang et al. unexpectedly find that 20-kDa myosin light-chain and myosin-11, important components of smooth muscle myosin II, are present at the edge of lamellipodia. This cellular localization of myosin II orchestrates the recruitment of a number of actin-related proteins to the leading edge and promotes ASM migration. Moreover, the authors uncover the mechanisms that control myosin II localization and activation.

Nestin is a type-VI intermediate filament protein, but its role in smooth muscle migration has not been previously investigated. Wang et al. use the state-of-art technology to seek answers to this interesting question. They report that nestin controls focal adhesion assembly and ASM migration through a previously unknown mechanism. Specifically, nestin regulates the activation of polo-like kinase 1, which catalyzes vimentin phosphorylation at Ser-56 and promotes the connection of vimentin filaments with focal adhesions and focal adhesion formation.

Asthma is clinically characterized as either eosinophil-high or eosinophil-low asthma. Eosinophil-high asthma typically is associated with higher Th2 cytokines. Interestingly, blood eosinophils can be described as inflammatory-like and lung-resident-like eosinophils. Palacionyte et al. describe that IL-5 and GM-CSF significantly enhance the proliferative effects of inflammatory-like and lung-resident-like eosinophils cells on ASM cells derived from patients with severe non-allergic eosinophilic asthma and individuals with non-severe allergic asthma. This study is interesting because the coculture of eosinophils and ASM cells affects ASM proliferation. Future studies are required to understand how this may occur.

Overall, these reviews and original research articles advance our knowledge regarding ASM biology and asthma pathobiology. However, there are still many unanswered questions. For instance, how may inflammation-independent asthma occur? What new roles may sex steroids play in asthma? Is dual pharmacological medicine clinically realistic? How are ASM migration and proliferation regulated? Answers to these questions may open new avenues to develop new therapies to treat asthma.
